# Statistical analysis on the incidence and predictors of death among second-line ART patients in public hospitals of North Wollo and Waghemira Zones, Ethiopia, 2021

**DOI:** 10.1038/s41598-024-60119-1

**Published:** 2024-05-13

**Authors:** Atitegeb Abera Kidie, Seteamlak Adane Masresha, Fassikaw Kebede Bizuneh

**Affiliations:** Department of Public Health, College of Health Science, Woldia University, Woldia, Ethiopia

**Keywords:** Incidence, Death, Second line ART, Treatment failure, Ethiopia, Diseases, Health care, Risk factors

## Abstract

Acquired immune deficiency virus, caused by the human immunodeficiency virus, is a significant global health concern. Sub-Saharan Africa particularly Ethiopia faces a high prevalence of human immunodeficiency virus. In low-income settings like Ethiopia, early mortality rates are elevated due to severe opportunistic infections and advanced disease at Anti-retroviral treatment initiation. Despite available treatments, delayed treatment initiation among Human Immunodeficiency Virus -infected individuals in Africa, including Ethiopia, leads to disease progression and increased mortality risk. This study aimed to identify the factors contributing to the death of HIV patients under treatment at second line regimen in public hospitals of North Wollo and Waghemira Zones. A retrospective cohort study with 474 patients was conducted in selected hospitals of North Wollo and Waghemira Zones. A parametric Weibull regression model was employed, and the adjusted hazard ratio served as the measure of association. Variables significantly affected the outcome of the study was determined at a p-value < 0.05, along with a 95% confidence interval for the variables. The patients were within the average age of 38.6(standard deviation ± 12.5) years and majority (45.57%) had no formal education. The overall death incidence rate among second-line anti-retroviral treatment patients was 1.98 per 100-person years [95% CI 1.4—2.9%]. Poor adherence to antiretroviral treatment, male gender, and being underweight significantly increased the hazard of death. Conversely, increased anti-retroviral treatment duration had a significant and negative impact, reducing the hazard of death among patients. The study reveals a high incidence of death among second line anti-retroviral treatment users. Independent predictors include poor adherence, male gender, and underweight status, all significantly increasing the risk of death. On the positive side, the hazard of death decreases with longer anti-retroviral treatment duration. A critical concern and counseling should be given for better ART adherence, to change their nutritional status and for males.

## Introduction

Acquired immunodeficiency syndrome (AIDS) was recognized as a new disease in 1981, marked by higher death rates among young homosexual men due to uncommon infections and rare cancers^1^. AIDS is an infectious disease caused by Human Immune Deficiency Virus (HIV), primarily targeting the immune system^2^. It's a major global health concern, with 25.8 million people living with HIV in Sub-Saharan Africa^3,4^. Ethiopia, similar to other Sub-Saharan nations, hosts one of the globe's largest populations of individuals living with HIV, with approximately 1.2 million infected individuals^5^. Despite ART progress and treatment-as-prevention efforts, around 2 million new HIV infections occur yearly^6^. Ethiopia is one of the most affected countries in Sub-Saharan Africa by the HIV epidemic, with an estimated 617,921 individuals living with HIV, where 78% of adults and children receive ART^7^. Ethiopia is severely impacted by the HIV pandemic, ranking among the most affected sub-Saharan African nations^8^. Second-line regimens are employed when first-line treatments fail, determined by factors such as the patient's CD4 cell count, HIV viral load, or clinical indicators^9^.

In low-income settings, early mortality rates were higher due to more severe opportunistic infections and advanced disease at ART initiation^10^. ART globally saved millions of lives and significantly reduced illness and death in people living with HIV/AIDS (PLWHA)^9^. HIV/AIDS remains a pressing issue, particularly in resource-limited nations, leading to significant morbidity and mortality^11^. Income Disparity Influences Mortality Rates in Adults on ART: Higher Mortality in Low-Income Countries. Mortality of adult patients who are on antiretroviral therapy (ART) is higher in low-income than in high-income countries^10^.

By the end of 2020, over 26 million individuals living with HIV globally had received antiretroviral therapy (ART), leading to a notable decrease in HIV-related illness and deaths^12^. Despite the considerable decrease in mortality and illness among individuals with HIV due to Highly Active Antiretroviral Therapy (HAART), some patients still experience mortality even after beginning ART^13^. Around 1.7 million individuals died from AIDS-related causes globally, with 70% of these occurrences happening in Sub-Saharan Africa, as per the 2012 UNAIDS report^8,14^.

ART's introduction linked to global reduction in HIV-Attributed Deaths. In resource-constrained settings, there has been notable high early mortality following the initiation of ART^15^. Despite available treatments, many HIV-infected individuals, notably in Africa like Ethiopia, delay treatment, leading to disease progression and higher risk of mortality^2^. Patients in resource-limited countries face a higher risk of mortality compared to those in high-income nations^16^. The government of Ethiopia has been working on the scaling up of ART for all people to reduce AIDS-related morbidity and death. Despite the scale-up of ART, early mortality is a major challenge. High rates of early mortality were reported from a number of Sub-Saharan African ART programs^17^. The advent of combination antiretroviral therapy (cART) significantly improved AIDS prognosis. However, discontinuation of ART in some HIV patients can contribute to adverse outcomes, including death^18^. Third-line regimens, being expensive and scarce in resource-limited settings, make second-line treatment the final available option for many patients in these regions^19^.

Limited access to treatment, poverty, and ignorance has all contributed to the elevated mortality rates associated with this illness in the area^20^. Most studies focus on baseline predictors. Although previous studies have identified the factors that influence mortality, it is important to note that these determinants can vary greatly depending on the context, setting and change over time. Research findings can be influenced by contextual factors or specific population characteristics. Moreover, cohort studies provide insights into the dynamics and trajectories of phenomena (death) and can help identify temporal patterns or causal relationships that may not be apparent in cross-sectional studies. This study aimed to identify the factors contributing to the death of HIV patients under treatment at second line regimen in public hospitals in North Wollo and Waghemira Zones. This can help identify the potential impact of contextual factors on the generalizability of research findings.

This may help programmers to tackle the cause of death through the cohort of HIV patients based in second line ART treatment. Studies are limited on the incidence and predictors of death during follow up period.

## Methodology

### Study design and setting

Retrospective cohort study was conducted in three hospitals of North Wollo and Waghemira Zones. The three hospitals included in this study were Woldia Comprehensive Specialized Hospital, St. Lalibella General Hospital, and Tefera Hailu Memorial Hospitals. These hospitals were selected because they are the only hospitals which provide second line ART.

### Sampling technique, inclusion and exclusion, sample size and data collection

This study was conducted based on secondary source data which was from patients medical records and charts.

A consecutive sampling method was applied and all HIV/AIDS patients who had followed up from September 2016 and April 2020 and who had minimum of 6 month on treatment of ART were included and incomplete charts were excluded. The patients who had followed up in the specified period was our study population. A total of 474 HIV/AIDS patients were used for this study.

Data extraction checklist was prepared based on literatures. An initial preliminary review was conducted to modify the extraction tool.

### Variables of the study

The outcome variable of this study was time from second – line ART initiation to death.

The independent variables included for this study was marital status, sex, age, education status, lost to follow up, opportunistic infections, BMI, functional status, duration of ART, WHO stage, virological failure, second line ART regimen, comorbidity, ART adherence and HIV disclosure and these variables were screened by bi-variable analysis.

### Operational definitions

#### Event

The event of interest for this study is the death of HIV/AIDS patients in second line ART treatment.

#### Censored

This study include those patients who are lost to follow up, live beyond the study period and deaths unrelated to their diseases.

#### ART adherence

The level of ART drug adherence was classified as good if ≥ 95% adherence by pill count, fair if 85% to 94% adherence by pill count, or poor if < 85% adherence by pill count.

### Data management and analysis

The collected data was entered by EpiData-3.3.1. The entered data was exported to Stata version 17 for further statistical analysis. According to the proportion of missing to be managed, missing data was managed by imputation method using R software.

Descriptive statistics was reported using text, table and graph. Specific to survival analysis, data were presented by Kaplan Meier survival curve. In this study survival analysis was done to identify predictors of incidence of death. Survival analysis is a statistical method used to analyze time-to-event data, where the event of interest is typically death, failure, or any other occurrence that marks the end of the observation period. In survival analysis, parameter estimation involves estimating the parameters of a survival model that describes the relationship between covariates and survival times. There are some commonly used parameter estimation methods for survival analysis. For parameter estimation, there are different survival models to be considered, these are semi-parametric (cox regression model) and parametric models (Exponential, Weibull, Gamma, Log normal,Loglogistic). The summary of formula of each parametric model is described as follows:

The commonly used parametric distributions and parameters:

**Table:** 

Distributions	f(t)	S(t)	h(t)
Exponential	λexp(-λt)	exp (− λt)	λ
Weibull	${\lambda pt}^{p-1}$ exp(${-\lambda t}^{p}$)	exp (${-\lambda t}^{p})$	${\lambda pt}^{p-1}$
Log-logistic	$\frac{{\lambda pt}^{p-1}}{{(1+{\lambda t}^{p})}^{2}}$	$\frac{1}{1+{\lambda t}^{p}}$	$\frac{{\lambda pt}^{p-1}}{1+{\lambda t}^{p}}$

where λ is the location parameter, p is shape parameter.

In this study, proportional hazard assumption was checked using graph, global tests and adding time varying covariates.

Model fitness was checked using AIC and a model with lower AIC value was selected as the best fitted model. Bi-variable analysis was done and variables with p-value less than 0.25 were entered into multivariable analysis. Finally to assess the association of incidence of death and its predictors among second line ART patients, a parametric weibull regression model was done. A measure of association using Adjusted hazard ratio (AHR) was used and the variable significantly affected the variable of the study was determined at a p-value < 0.05, along with a 95% confidence interval for the variables.

### Ethics approval and consent to participate

To conduct the study, ethical approval was obtained from Woldia University institutional review board (WDUIRB). Since the study was secondary data and which was not directly obtained from patients, permission letter to access the charts and records was given for selected hospitals of North Wollo and Waghemira zone.

## Result

### Socio- demographic characteristics of participants

Regarding their demographic status, almost half 238(50.21%) of HIV/AIDS patients were in the age group 41 and above years old. Majority had no formal education (45.57%) and few (18.35%) were attended secondary and above education. Males were the dominant HIV/AIDS patients on second-line ART in this study which accounts 54.85%. Near to half of the patients were married (46.2%) (Table1).

**Table 1:** Socio-demographic characteristics of HIV/AIDS patients on second-line ART in North Wollo and Waghimra zone public hospitals.

Variables	Category	Frequency	Percent
Age	< 18	42	8.86
	18–40	194	40.93
	41 and above	238	50.21
Educational status	No education	216	45.57
	Primary	171	36.08
	Secondary and above	87	18.35
Residence	Urban	287	60.55
	Rural	187	39.45
Marital status	Single	139	29.32
	Married	219	46.20
	Divorced	116	24.47
Religion	Orthodox	390	82.28
	Other	84	17.72
Sex	Male	260	54.85
	Female	214	45.15

### HIV related and other clinical important variables

Most 406 (85.65%) of patients had working functional status. Around 185 (39.03%) were underweight, 261 (55.06%) normal and 28 (5.91%) overweight/obese. Majority were in WHO T-stage one which accounts 358 (75.53%) and 26 (5.49%) had lost to follow up. There was considerable magnitude of virological failure which accounts about 73 (15.40%). Majority 393 (82.91%) had good adherence and almost all 316 (91.59%) disclose their HIV status (Table 2).

**Table 2:** HIV related and other clinical important variables of HIV/AIDS patients on second- line ART in North Wollo and Waghemira zone public hospitals.

Variables	Category	Frequency	Percent
Functional status	Ambulatory/bedridden	68	14.35
	Working	406	85.65
BMI	Underweight	185	39.03
	Normal	261	55.06
	Overweight/obese	28	5.91
WHO T stage	T1	358	75.53
	T2	70	14.77
	T3 and T4	46	9.70
Lost to follow up	Yes	26	5.49
	No	448	94.51
Second line virological failure	Yes	73	15.40
	No	401	84.60
Comorbidity	Yes	34	7.17
	No	440	92.83
ART adherence	Good	393	82.91
	Moderate	33	6.96
	Poor	48	10.13
HIV disclosure	Disclosed	316	91.59
	Not disclosed	29	8.41

### Kaplan–Meier estimates

**Figure 1:**
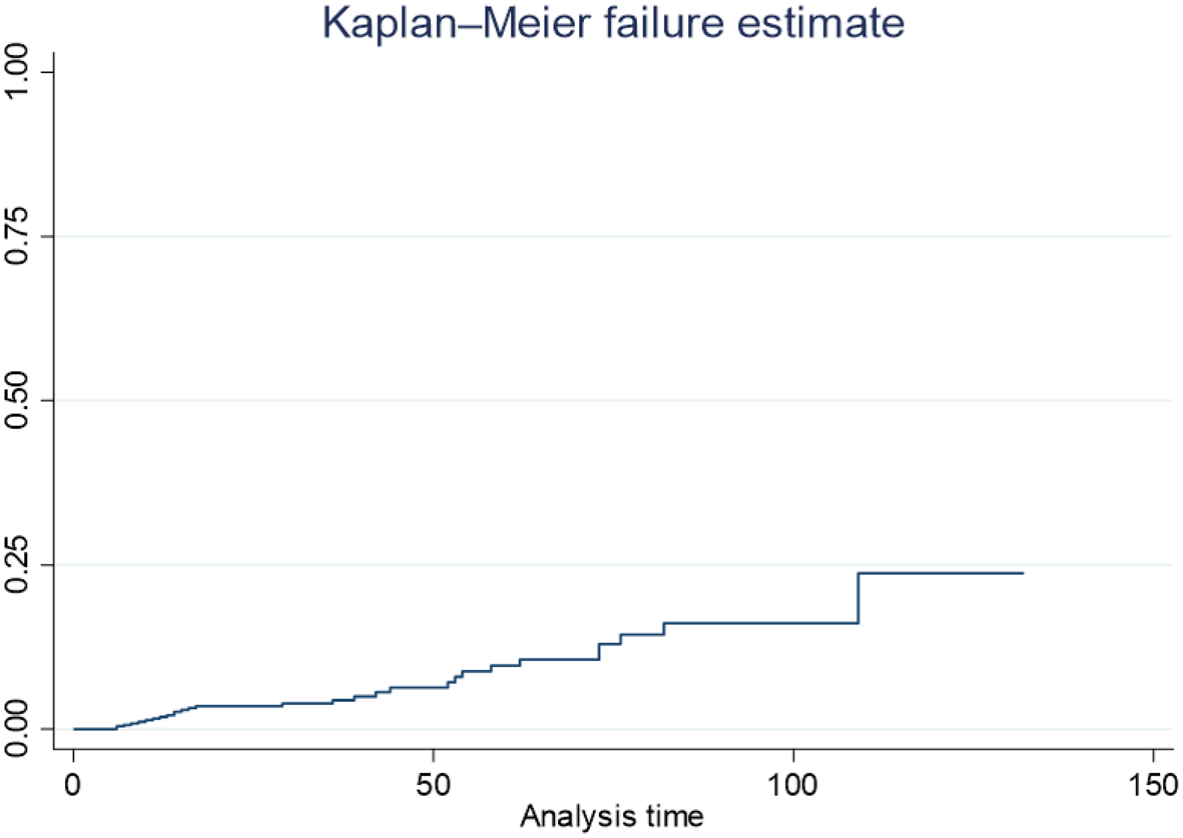
Cumulative hazard of death of second line ART patients in North Wollo and Waghemira zone public hospitals.

The Kaplan-Maier curve provides a useful summary of survival data and can be used to estimate median survival time. The curve showed that the data had no median survival time. This indicated that the number of censored is greater than event of interest (Fig. 1).

### Incidence of death

There were 29 deaths and the person time of death was 1461.5 years. The overall incidence rate of death among second line ART patients were 1.98 [95% CI of 1.4 & 2.9%] per 100- person year.

### Factors associated with incidence of death

Initially cox proportional hazard assumptions were assessed. The graph showed that the assumption was violated. In order to confirm the violation of the assumption, a global test was used (phtest) and indicated that there was enough evidence to reject the hypothesis of proportional hazard assumption at p-value less than 0.05 (Global test p- value = 0.049). In addition, the information criteria support that the proportional hazard model was not the best fitted model. Therefore, parametric models were considered such as exponential, Weibull, Log normal, Log logistic and Gompertz.The best fitted model was parametric Weibull model and selected using information criteria such as log likelihood ratio test, AIC and BIC (Table 3).

**Table 3:** Model comparison for selection of the best fitted model.

Models	LLR	AIC	BIC
Exponential	− 100.6	223.1	268.9
Weibull	− 95.2	214.3	264.3
Log normal	− 98.6	221.2	271.2
Log logistic	− 96.3	216.6	266.5
Gompertz	− 95.7	215.5	265.4

*LLR* logliklihood ratio, *AIC* Akake information criteria, *BIC* Bayesian information criteria.

Lastly, multivariable Weibull regression model was used to assess the predictors of death. In multivariable Weibull regression, ART adherence, ART duration and BMI were significantly associated with incidence of death among second line ART patients. Patients who had poor ART adherence were 3.59 times more hazard of death as compared to those with good adherence [AHR = 3.59, 95% 1.39,9.24]. Sex of patients was significantly associated incidence of death, being male increased the hazard of death by 2.36 times as compared to males [AHR = 2.36, 95% CI 1.06, 5.29]. Regarding duration of ART, increasing the duration of ART by one year decreases the hazard of death by 0.97 times [AHR = 0.97, 95% CI 0.96, 0.98]. Patients who are underweight had more hazard of death by 3.12 times as compared with those normal BMI [AHR = 3.12, 95% CI 1.37, 7.12] (Table 4).

**Table 4:** Predictors of death of HIV patients under treatment at second- line regimen in North Wollo and Waghemira zone public hospitals.

Variables	Categories	CHR (95% CI)	AHR (95% CI)	P-value
ART adherence	Good	1	1	1
	Moderate	0.78(0.10, 5.85)	0.83 (0.11, 6.40)	0.879
	Poor	5.06(2.34, 10.96)	3.59 (1.39, 9.24)*	**0.015**
HIV disclosure Status	Disclosed	1	1	1
	Not disclosed	2.37(0.82,6.83)	2.48 (0.81, 7.53)	0.097
Sex	Female	1	1	1
	Male	1.90(0.89, 4.03)	2.36 (1.06, 5.29)*	**0.033**
ART duration		0.98(0.96, 0.99)	0.97(0.96, 0.98)*	** < 0.0001**
Virological failure	No	1	1	1
	Yes	1.78(0.73, 4.40)	1.24 (0.48, 3.20)	0.650
BMI	Normal	1	1	1
	Underweight	2.28(1.06, 4.92)	3.12 (1.37, 7.12)*	**0.011**
	Overweight/obese	1.37(0.30, 6.20)	0.86 (0.16, 4.52)	0.844
WHO T stage	T1	1	1	1
	T2	0.39(0.09, 1.69)	0.34 (0.07, 1.54)	0.167
	T3 & T4	2.23(0.99, 5.03)	1.14 (0.44, 2.96)	0.758

Significant values are in bold.

*CHR* crude hazard ratio, *AHR* Adjust hazard ratio, *CI* Confidence interval, *1* reference.

*p -value < 0.05.

## Discussion

This study was aimed to identify the predictors of incidence of death among second line ART patients. Around two present of patients were died during their second line ART follow up. This finding was lower than a study finding of Tanzania, Dessie, Metema, Amhara region, Gofa Zone of SNNPs, Aksum^4,10,12,13,21,22^. This study was in lined with a study conducted in Harari hospital Ethiopia^16^. This study finding was also below the study result of Nekemte, Gondar and Debre Markos Comprehensive Specialized Hospital^23–25^. The possible variation might be the difference in study sample size or denominator and the characteristics of study participants in relation to socio demographic, socio economic and cultural variations.

Regarding predictors, those patients who had poor ART adherence were significantly and positively associated with the risk of death. This is in line with a study conducted in selected hospitals of Amhara region and Dessie referral hospital^12,21^. Other studies also support this finding like studies conducted in Harar, Africa and Asia point out that HIV-infected patients with poor ART adherence had a higher risk of death as compared to those who had good ART adherence^16,19^.Similarly a study conducted in West Amhara, Gofa zone of SNNPs, Tanzania and Sub-Saharan African Sites showed that poor ART adherence had the highest risk of death than adherent patients^2,22,26,27^. Study conducted in Nekemte showed that poor ART adherence was found to be an independent predictor of death^25^. However, in contrary to the study finding conducted at Gondar that fair adherence level had high hazard of death^23^. The reason might be because poor adherence leads to low levels of drug, driving to viral replication, drug resistance, and viral rebound. That in turn causes low CD4 count which leads to morbidity and death^28^.

Being male also significant predictor and increased the incidence of death of HIV/AIDS patients on second line ART. This finding is in line with the study conducted in Uganada, Iran and Zambia^29–31^. However this is in contrast with the study finding of Tennessee^32^. Other study conducted in China and Nepal showed that men have a significantly higher risk of death than female^33,34^. There is no scientific evidence on the mechanism how males are more prone to risk of death than females. But different studies point out possible reasons for their death as males have less health care access than female might be due to high workloads. Males delay in treatment adherence and may not have better recovery of immune system as compared to females^35,36^.

Increasing ART duration by one year reduced the risk of incidence of death during their follow up. This finding is comparable with the study conducted in southern India^37^. This is also supported by the study conducted in Debre Tabor and Southern Oromiyaa region indicated that the death rate of patients in the earlier months of ART initiation was high and it declined in the later months of follow-up^20,38^. The study conducted in Korea and Nepal showed that the risk of death was lower in late ART follow up (1–5 years later) than the first year of ART duration which is similar to this study finding^34,39^. The explanation could be that with a longer duration of antiretroviral therapy (ART), there is a potential for an increase in CD4 count, improved viral suppression, and a reduced likelihood of co-infection compared to the early stages of ART initiation. In the initial phases of ART, there might not be significant improvements in patient immunity, making them more susceptible to infections. However, as the duration of ART progresses, these factors may diminish, leading to a lower risk of death.

Nutritional status is the other key significant predictor that those patients who are underweight face the risk of death as compared to those normal BMI range. This finding was supported by the study conducted in Metema, Tanzania, East Africa and Nigeria, Sub-Saharan African Sites and found that being underweight patents were at high risk of death^8,13,26,40^. Being underweight increases the likelihood of negative clinical results and serves as a predictor for the development of opportunistic infections (OIs) in individuals with HIV/AIDS, ultimately leading to death^41^.

## Conclusion

The incidence of death among HIV/AIDS patients on second line ART was high. The risk of death was significant among patients with poor adherence and those who are male and underweight. However, the hazard of death decreased as the duration of ART increased. Therefore, a critical concern and counseling should be given for better ART adherence, to change their nutritional status and for males.

### Strengths and limitations of the study

As compared to cross-sectional survey, this study has strengths related to the design. This study was conducted by retrospective cohort design. Therefore, temporal relationships can be determined for the outcome variable and its predictors. Even though this study had strengths,there was limitations related to the source which was secondary nature of the data. Since the data was from patient records and charts, some important variables were not available.

## Data Availability

The data used for this study is available in the manuscript. It also available at the hand of all authors and can be given upon reasonable request.

## Abbreviations

AHR: Adjusted hazard ratio
ART: Anti-retroviral treatment
BMI: Body mass index
HAART: Highly active antiretroviral therapy
HIV: Human immunodeficiency virus
OIs: Opportunistic infections
SNNPs: South Nations Nationalist and People
WHO: World health organization
